# Immune signatures predicting the clinical outcome of peanut oral immunotherapy: where we stand

**DOI:** 10.3389/falgy.2023.1270344

**Published:** 2023-10-02

**Authors:** Naphisabet Wanniang, Theresa-Maria Boehm, Françoise Codreanu-Morel, Amandine Divaret-Chauveau, Isabela Assugeni, Christiane Hilger, Annette Kuehn

**Affiliations:** ^1^ Department of Infection and Immunity, Luxembourg Institute of Health, Esch-sur-Alzette, Luxembourg; ^2^ Faculty of Science, Technology and Medicine, University of Luxembourg, Esch-sur-Alzette, Luxembourg; ^3^ Department of Allergology and Immunology, Centre Hospitalier de Luxembourg-Kanner Klinik, Luxembourg, Luxembourg; ^4^ Pediatric Allergy Department, Children’s Hospital, University of Nancy, Vandœuvre-lès-Nancy, France; ^5^ EA3450 DevAH, Faculty of Medecine, University of Lorraine, Vandoeuvre-lès-Nancy, France

**Keywords:** oral immunotherapy, peanut allergy, immune response, immunophenotyping, immunotherapy, food allergy

## Abstract

Peanut allergy is a growing health concern that can cause mild to severe anaphylaxis as well as reduced quality of life in patients and their families. Oral immunotherapy is an important therapeutic intervention that aims to reshape the immune system toward a higher threshold dose reactivity and sustained unresponsiveness in some patients. From an immunological point of view, young patients, especially those under 3 years old, seem to have the best chance for therapy success. To date, surrogate markers for therapy duration and response are evasive. We provide a comprehensive overview of the current literature state regarding immune signatures evolving over the course of oral immunotherapy as well as baseline immune conditions prior to the initiation of treatment. Although research comparing clinical and immune traits in the first years of life vs. later stages across different age groups is limited, promising insights are available on immunological endotypes among peanut-allergic patients. The available data call for continued research to fill in gaps in knowledge, possibly in an integrated manner, to design novel precision health approaches for advanced therapeutic interventions in peanut allergy.

## Introduction

### Peanut allergy: a growing health concern

Food allergies can be IgE-mediated, non-IgE-mediated, or mixed types (1–3). IgE-mediated peanut allergy (PA) is one of the most common food allergies in both children and adults (4, 5). PA is also associated with severe reactions and a poor prognosis of outgrowth (10%–27% resolution at 4–12 years of age) (6, 7). PA appears to have the highest prevalence rate of 1%–2% in the Western population (4). In the United States, PAs increased 3.5-fold between the years 1997 and 2008 (8), which might vary in other regions (9). PA negatively affects the quality of life of patients and their caregivers, contributing to a significant financial and healthcare burden for society (4, 8, 10).

With the advent of immunotherapy options, monitoring therapy success by dissecting changes from a clinical and immunological point of view is strongly needed. Here, we review the present evidence and knowledge gaps for stratifying patients based on immune signatures before starting treatment in different age groups using oral immunotherapy (OIT).

### Pathophysiology: disturbed epithelial barrier to Th2 immune skewing

PA results from an immune dysregulation to peanut proteins, with cupins (Ara h 1, Ara h 3) and 2S albumins (Ara h 2, Ara h 6) being highly potent allergens (11). Sensitization to such antigens occurs via damaged epithelial barriers (mainly the skin and gut), resulting in a release of epithelium-derived cytokines, interleukin (IL)-25, IL-33, and thymic stromal lymphopoietin (12). These “alarmins” activate dendritic cells (DCs), which captures allergens, inciting the expression of OX40L. OX40l then binds to OX40 on naïve T cells, skewing the immune system toward a T-helper type 2 (Th2) inflammatory response and producing IL-4, IL-9, and IL-13 (13–15). Apart from Th2 cells as a hallmark of allergic inflammation, innate lymphoid cells (ILC2) increase in number and secrete proinflammatory cytokines, such as IL-4, IL-5, and IL-13, which affect Th2 differentiation and activate B cells and effector cells [mast cells (MCs), basophils, and eosinophils] (16, 17). IL-4, secreted by antigen-specific Th2 cells, prompts class switching of B cells, resulting in the production of peanut-specific IgE (sIgE) antibodies. How Th2A cells, a Th2 subpopulation with low proliferation capacity, are involved in IgE production requires further clarification (18). T follicular helper (Tfh) cells producing IL-13 (Tfh 13) appear to further regulate the production of high-affinity sIgE. sIgE binds to the high-affinity IgE receptor (FcεRI) on the surface of effector cells such as MCs and basophils (3, 12, 19).

Upon re-exposure to peanuts, cross-linking of the sIgE bound to effector cells results in cell degranulation. This process induces the release of mediators such as histamine, prostaglandins, tryptase, and platelet-activating factors responsible for clinical manifestations of PA (20). Other immune processes further reinforce allergic inflammation. In a feedback loop mode, CD209^+^ monocyte-derived DCs increase the frequency of peanut-specific CD4^+^ T cells to enhance persisting PA responses (21).

### Clinical assessment: understanding variable patterns of clinical reactivity

PA may present with mild symptoms to severe anaphylactic reactions. According to the Allergy Vigilance Network and European anaphylaxis registries, peanut-induced anaphylaxis is mainly common in children (15.4% in 2007–2018; 20% in 2002–2020) (22, 23). Intrinsic and extrinsic factors, such as the clinical history of asthma or previous reaction, sensitization to Ara h 2/Ara h 6, and the presence of cofactors (e.g., exercise and acute infections), may increase the risk of anaphylaxis (23, 24). Doses eliciting objective symptoms in 10% of PA patients appear low, ranging from 2.8 to 6.6 mg of peanut protein (25). However, threshold dose reactivity varies greatly among patients (26, 27). This variability might be due to multiple reasons, including the presence of cofactors, dietary composition, and digestion kinetics (28). A subgroup of patients experiences allergic cross-reactions to tree-nuts and botanically related legumes, such as lentil, lupine, and pea (17, 29–31). Typically, diagnosing PA is straightforward by combining the clinical history of the patient with tests involving peanut extract-sIgE and/or skin tests [95% positive predictive value (PPV): sIgE ≥ 35 kU_A_/L at 1 year of age, ≥2.1 kU_A_/L at 4 years; skin test wheal ≥8 mm] (32–34). Using single allergens for sIgE quantification further enhances the diagnostic accuracy (e.g., Ara h 2 ≥ 42.2 kU_A_/L in children between 2 and 7 years with PPV >95%) (35). Although often limited to research settings, the basophil activation test (BAT) can also be used in diagnosing PA; however, oral food challenges (OFCs) continue to be the gold standard (36, 37).

## Immune signatures in OIT

### Immunotherapy in clinical practice: treatment via the oral route

**Figure 1:**
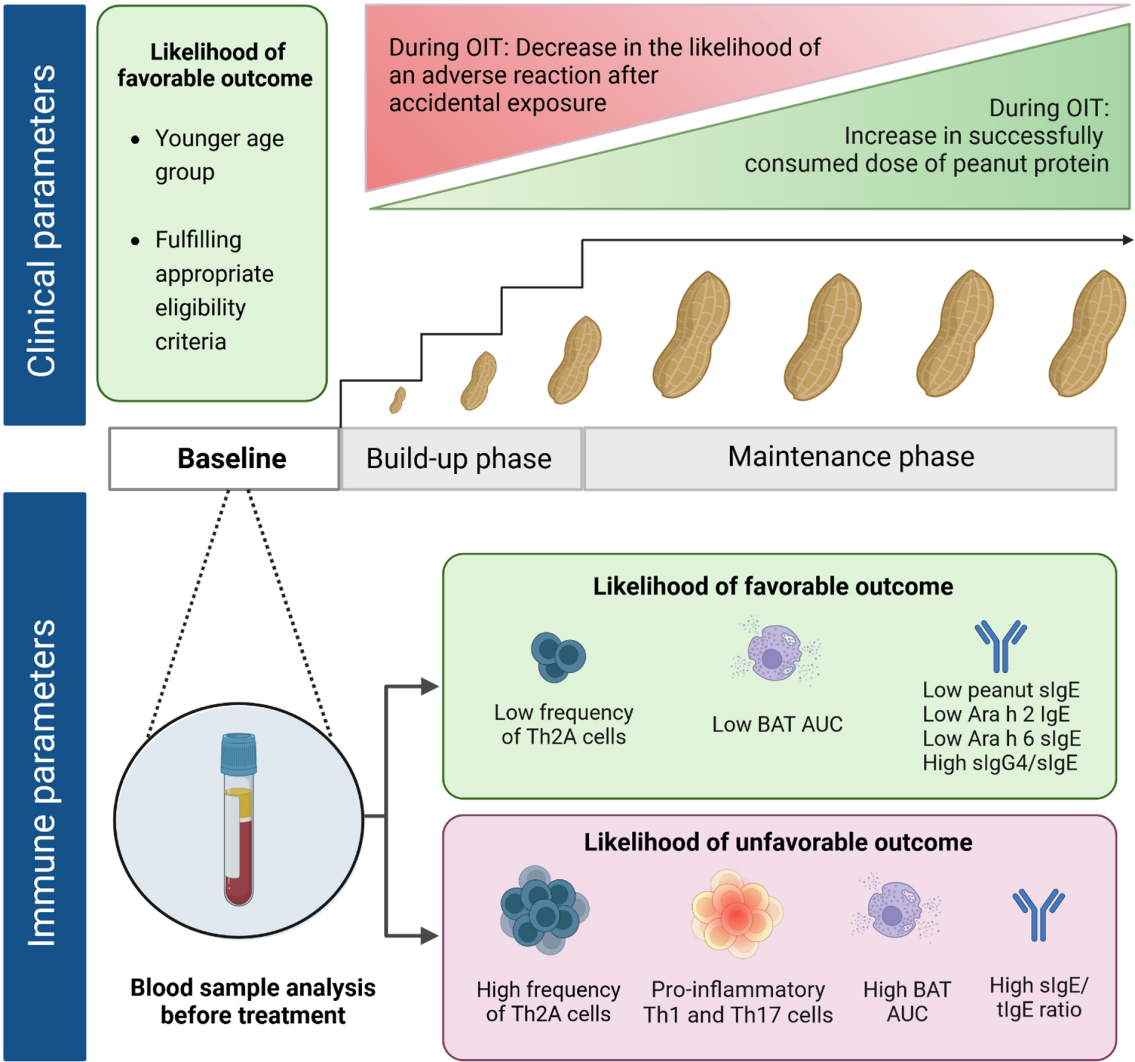
Clinical and immune parameters at baseline correlating with treatment outcomes of peanut oral immunotherapy. Favorable outcome, i.e., desensitization (ability to successfully complete the build-up phase without adverse reactions requiring epinephrine and reach a set maintenance dose OIT or able to successfully pass an exit OFC) and/or sustained unresponsiveness (ability to pass an exit OFC even after OIT discontinuation). Appropriate eligibility criteria, e.g., good compliance and absence of severe/uncontrolled asthma.

Managing PA based on strict avoidance is a strategy that leaves patients with a reduced quality of life and an increased risk of severe reactions to accidental exposures (38, 39). While therapeutic treatments with biologicals are under investigation in clinical trials (e.g., monoclonal anti-IgE antibody), OIT is presently the most commonly used treatment (40). Other alternative routes of immunotherapy (e.g., sublingual and epicutaneous) also show promising results in clinical studies (41, 42). Based on the extensive literature available on the efficacy of OIT, the Global Allergy and Asthma European Network (GA^2^LEN) recommends OIT for selected PA patients >4 years of age (43). The first OIT treatment using peanut powder was approved recently (44). Other OIT trials and real-life clinical practice often depend on commercially available food-grade products (e.g., peanut flour or snacks) (45–47). Overall, clinical outcomes can be highly variable. Through gradually increasing and continued intake of peanut doses, OIT increases the level of threshold dose reactivity as long as patients continue OIT (“desensitization”) (48, 49). Efficacy in achieving desensitization appears high (>60%–90%), as reported for children >3 years, adolescents, and adults (44, 45, 50). A state of remission, also often referred to as sustained unresponsiveness (SU), reported after a few months of OIT discontinuation, is seen only in a small subset of patients (51). Earlier trials on OIT used a higher maintenance dose of up to 4,000 mg of peanut protein daily, while recent studies have demonstrated similar efficacy with a lower maintenance dose of 300 mg (47, 50, 52–54). However, the exact duration and dose of OIT required to achieve long-term remission are still unclear (50–53). SU seems to develop more easily during OIT in children under 4 years. Both the IMPACT and DEVIL studies revealed a higher SU rate in younger age groups, 71% (<2 years) and 78% (<3 years of age), respectively (51, 54). From an immunological point of view, such a young patient group seems to be the ideal candidate to start OIT. However, because of safety concerns, the potential for spontaneous resolution, and logistic constraints, the GA^2^LEN recommends OIT at this age based on individual profiles (43). Thus, a careful patient-centered evaluation remains a crucial prerequisite to OIT (55–58). The availability of surrogate markers would be an important asset in refining patient selection for OIT in the future (Figure 1).

We refer to available reference literature for details on the immune mechanisms during OIT (16, 59, 60). Below, we outline key events of immune changes and integrate these with baseline conditions for different age groups (Table 1).

**Table 1:** Immune signatures correlating with OIT outcomes in peanut-allergic patients.

Age	Biomarker	Biomarker at OIT baseline	Biomarker over OIT course	Correlation with immunotherapy outcome	Reference
Cellular markers					
Infants/toddlers	Th2A cells	CD154^+^ and CD137^+^ pTeff expressing CRTH2^+^CCR6^−^CXCR5^−^ in PBMC following *ex vivo* stimulation	N/A	Desensitized patients had a Th2A-high immunotype at baseline (>20% of pTeff) while patients who achieved SU had a Th2A-low immunotype (<20% of pTeff)	(18)
				PA reactors (positive DBPCFC at ≤100 mg peanut protein) have a higher frequency of Th2A^+^ pTeff cells compared with non-reactors at baseline	
Children (older than 4 years) to adults		N/A	GATA3, IL17RB, and PTGDR2 expression in isolated PBMC by single-cell RNA-seq	Lack of suppression of Th2A cells (determined by decrease in Th2 gene module expression) at maintenance OIT (12 weeks after maximum dose was reached) was associated with treatment failure	(61)
				Failure is defined as not achieving the minimum maintenance dose (600 mg) of peanut protein by 12 months, an ED < 1,443 mg at DBPCFC2, ED at DBPCFC3 < 443 mg, or <10-fold more than that at DBPCFC1	
	Th1, Th17 cells	Increased expression of OX40, OX40L, STAT1, Th1, and Th17 gene modules by the transcriptomic approach	N/A	Baseline non-Th2 inflammation was associated with treatment failure	(61)
	Basophil	% of CD63high PE AUC in whole blood	N/A	Basophil “non-responders/low responders” (peanut-induced %CD63^high^ dose–response AUC < 12.09) had better treatment outcomes, where 83% and 33% of patients who discontinued OIT after 2 years passed OFC up to 4 g of peanut protein at weeks 117 (∼3 months after OIT discontinuation) and 156 (1 year post-OIT), respectively. When considering patients who continued OIT at a maintenance dose of 300 mg daily, 91% and 55% passed OFC at weeks 117 and 156, respectively.	(62)
				Only 17% basophil “high responders” (peanut-induced %CD63^high^ dose–response AUC > 97.37) passed OFC at weeks 117 and 156.	
		N/A	AUC to Ara h 2 by indirect basophil activation assay	Sensitivity of basophils to Ara h 2 significantly reduced as early as 3 months of OIT and persisted even post-OIT discontinuation in patients who attained SU (passed 5 g of peanut protein OFC after 4 weeks of OIT avoidance).	(63)
				For patients who were only transiently desensitized (failed OFC after 4 weeks of OIT discontinuation), this early change during OIT was unobserved, and they had a significant rebound in their basophil AUC when OIT was discontinued.	
	Myeloid DC	N/A	TNF-α-producing HLADR^+^CD11c^+^ mDC in PBMC following *ex vivo* stimulation	OIT resulted in a decreased frequency of these DCs in “OIT responders” (defined as a successful build-up phase without adverse reactions requiring epinephrine and achieved maintenance dose), while “non-responders” showed an increase in these cells.	(64)
Serological markers					
Preschool toddlers	Peanut-sIgE	<35 kU_A_/L	N/A	93% (25/27) of the patients attained SU (defined as lack of reactivity to a challenge with 5,000 mg of peanuts after 4 weeks of OIT discontinuation).	(51, 65)
	Ara h 2-IgE	<24 kU_A_/L	N/A	93% (25/27) of the patients with Ara h 2 sIgE below the cut-off attained SU	(50, 65)
	sIgE and Ara h 2 sIgE	<35 kU_A_/L and <24 kU_A_/L, respectively	N/A	Taking these cut-offs together, the PPV of attaining SU was 0.93	(66)
	Ara h 6-IgE	Component-specific IgE	N/A	Lower component sIgE to Ara h 6 predicted desensitization (passed DBPCFC to 5 g peanut protein after 134 weeks of OIT)	(51)
Children to adults	Peanut-sIgE	Extract-specific IgE	N/A	Lower peanut-sIgE correlated with successful OFC at week 117 in both peanut-0 (discontinued OIT after 2 years of active intake) and peanut-300 (after 2 years of active intake, continued with low MD of 300 mg/daily) groups.	(50, 65)
				Lower peanut-sIgE predicted remission (passed DBPCFC to 5 g peanut protein after 26 weeks of OIT discontinuation).	
	Ara h 2-IgE	Component-specific	N/A	Lower Ara h 2-sIgE correlated with successful OFC at week 117 in both peanut-0 (discontinued OIT after 2 years of active intake) and peanut-300 (after 2 years of active intake, continued with low MD of 300 mg/daily) groups	
	sIgE/total IgE	Extract-specific IgE and total IgE	N/A	Higher risk of treatment failure with high sIgE/total IgE ratio at baseline	(50)
	sIgG4/sIgE	Extract-specific IgE and IgG4	N/A	A higher ratio was significantly associated with successful OFC at week 117 in the peanut-0 group (discontinued OIT after 2 years of active intake).	(50)

PE, peanut extract; PBMC, peripheral blood mononuclear cell; N/A, not available.

### Immune changes occurring through OIT in children and adults

#### Th2 pathway

Early during OIT, CD63^+^ or CD203c^+^ basophil activation capacity decreases (62, 67). This rapid threshold increase in effector cell activation correlates with clinical desensitization and decreased skin test wheal diameter (59). Simultaneously, an initial peanut-specific IgE increase occurs during the build-up phase (51, 55). Expanding peanut-specific Th2 cells and memory B cells most likely contributes to the transient increase in peanut-sIgE (59, 68). During the maintenance phase, sIgE levels slowly decrease. This decline persists with ongoing treatment, as reflected by peanut-sIgE to total IgE (tIgE) ratios (69–71). As immunotherapy progresses, there is a reduction in the proliferative capacity of the pathogenic Th2 cells, along with a selective deletion of these cells and a decrease in the production of Th2 cytokines (IL-3, IL-5, and IL-9) (3). Such a downregulation appears to occur at both low (300 mg) and high (3,000 mg) maintenance doses (72). Indeed, this downregulation also correlates with clinical effects, as reported in the Probiotic Peanut Oral Immunotherapy (PPOIT) study based on reprogramming Th2 gene networks in the CD4^+^ T cell compartment (73). Expanding IL-10-producing regulatory T cells (Tregs) or B cells (Bregs) may be responsible for coordinating these events (59). IL-10 may also play a role in the induction of allergen-specific sIgG4, another hallmark of OIT (3, 44), as well as in other routes of allergen-specific immunotherapy (74–76).

#### Non-Th2 pathways

Manohar et al. showed that omalizumab-facilitated multi-OIT was able to not only decrease the frequency of peanut-reactive IL-4^+^CD4^+^ T cells but also modulate CD8^+^ T cells, γδ T cells, and patterns of homing marker expression. The expression of homing receptor G protein-coupled receptor 15 (GPR15) was downregulated on CD4^+^CD8^+^ and γδ T cells on treatment with omalizumab, which persisted when multi-OIT was continued. Skin homing markers such as C-C motif chemokine receptor type 4 (CCR4) and cutaneous leucocyte-associated antigen receptor (CLA) were upregulated on CD4^+^ and CD8^+^ T effector memory cells, respectively. The authors hypothesized that omalizumab-facilitated OIT could modulate effector T cell trafficking to target tissues (77).

#### Innate immune response

Zhou et al. demonstrated the role of natural killer (NK; CD56^+^, CD16^+^) cells in PA. Following OIT, fewer activated CD69^+^ NK cells were expressing both Th2 and Th1 cytokines upon peanut stimulation, which reduced significantly at the end of the maintenance phase compared with the baseline (66). In a small group of six PA children, OIT deviated the positive feedback loop between CD209^+^ monocyte-derived DCs and peanut-specific T cells, indicating the termination of PA responses (21).

#### Changes over OIT—infant/toddler vs. later in life

The detailed immune changes during OIT reported above were observed in patients older than 4 years. As infants and toddlers have a much better SU rate than older children, corresponding data are urgently needed to link their functionally distinct and uniquely regulated immune response to an improved OIT outcome (78).

### Immune characteristics at OIT baseline linked to different age groups

#### Baseline immune cell profile

#### Heterogeneity of antigen-specific CD4^+^ T cells

Repertoires of peanut-reactive effector CD154^+^ T cells (pTeff) might vary among patients (79). Monian et al. (61) described the baseline characteristics of peanut-reactive CD4^+^ T cells and their correlation with clinical responses in PA patients aged 7 years and older. Six subtypes of highly clonal peanut-reactive pTeff were identified, namely, three Th2 phenotypes (Tfh2-like: high in costimulatory marker, CXCR5 and PDCD1 gene expression, resembling Tfh13 subsets; Th2reg-like: FOXP3 and TNFRSF9; and Th2A-like: GATA3, IL17RB, and PTGDR2), two Th1 phenotypes (Tfh1-like and Th1-conventional/conv), and one Th17 subtype. Lack of Th2A-like suppression during OIT positively correlated with poor treatment outcomes. In addition, a high degree of baseline inflammation seen by increased expression of inflammatory gene signatures on Th1 and Th17 cells (OX40, OX40L, STAT1, IL-17, and GPR15 genes) was associated with immunotherapy failure, indicating that an existing non-Th2 inflammation could limit successful OIT (61). In line with these findings, Calise et al. (18) confirmed pTeff baseline responses in the IMPACT trial, with PA children less than 4 years of age. Using *ex vivo* T cell profiling and flow cytometry, they described three main subtypes that were stable in the absence of OIT, namely, conventional Th2 (CRTH2^−^CXCR5^−^CXCR3^−^CCR6^−^CCR4^+^), Th2A (CRTH2 ^+ ^CCR6^−^), and Th17-like (CRTH2^−^CCR6^+^).

#### Antigen-specific Th2 CD4^+^ T cell subsets

Based on previous findings, Berin et al. collectively described peanut-reactive CD4^+^ T cells as “Type 2 cells” encompassing Th2, Th2A, and Tfh13 subsets based on CD154 positivity and co-expression of IL-4 or IL-13 in PA patients from the Consortium for Food Allergy Research (CoFAR6 cohort) trial (80–82).

In PA patients aged 4 years and older, Bajzik et al. reported the role of Th2A pTeff in a subset of 42 patients from the PALISADE trial. Baseline conditions of patients with a low threshold dose reactivity (≤100 mg peanut protein) show a high frequency of pTeff cells predominantly of the Th2A phenotype (CRTH2^+^ pTeff). These Th2A cells also correlated with initial sIgE (44, 83). Luce et al. supported the selective suppression of those cells and showed similar findings under omalizumab-facilitated multi-OIT in 42 patients aged 5 years and older (here, the Th2A cell phenotype is CD4^+^CD45RO^+^CD45RB^−^CD27^−^CD49d^+^CD161^+^) (84). In PA children less than 4 years of age, the investigators of the IMPACT trial demonstrated that low baseline levels of Th2A pTeff cells were associated with SU after OIT completion (18). PA patients were classified as “Th2A-high immunotype” if Th2A subsets were >20% of total pTeff or “Th2A-low” if Th2A is <20% of pTeff. The Th2A-high immunotype is related to higher sIgE, sIgG4, and pTeff compared with the Th2A-low group. During active OIT, there was a selective decrease in the frequency of Th2A cells in all patients. Importantly, desensitized patients had a Th2A-high immunotype at baseline, while patients who achieved SU had a Th2A-low immunotype (desensitization refers to being able to tolerate 5 g of peanut protein by week 134 without severe symptoms; meanwhile, SU means remaining desensitized despite 26 weeks of OIT discontinuation).

#### CCR6^+^ peanut-reactive effector CD154^+^ T cell subset

In a subset of PA patients with low sIgE values at baseline, Th17-like CCR6^+^ pTeff appear to be the dominant immunotype, with inverse correlation to Th2A pTeff frequency (18, 81, 83). Bajzik et al. also showed that non-reactors [negative double-blind placebo-controlled food challenge (DBPCFC) at ≤100 mg peanut protein] had higher circulating CCR6^+^ pTeff at baseline compared with PA individuals with lower threshold dose reactivity. A transcriptomic analysis of these cells revealed the expression of Th1/Th17 and Treg-related genes (IFN-γ, RORγt, IL-17A, IL-17F, IL-22, IL-23R, CCL20, and FOXP3) (83). Reports on whether OIT affects this cell subtype are conflicting. In the PALISADE cohort, OIT did not modulate the frequency of this T cell subset, while in a multi-OIT study with omalizumab, the frequency of Th17 cells (CD4^+^CCR4^+^CCR6^+^) was reduced at week 30 (84).

#### TNF-α-producing dendritic cells

In PA children aged 5–12 years, OIT “responders” (defined as a successful build-up phase without adverse reactions requiring epinephrine and achieved maintenance dose) exhibited a decreased frequency of TNF-α-producing myeloid DC (mDC; defined as HLADR^+^CD11c^+^) (64). On the other hand, “non-responders” (defined as children with ≥1 adverse event requiring epinephrine and not reaching set maintenance doses) showed an increase in TNF-α-producing mDC. Moreover, responders had a transient reduced OX40L expression on mDCs at week 18 of OIT, contrary to non-responders. These findings suggested that a reduced proinflammatory DC phenotype in DCs could potentially contribute to predicting OIT outcomes.

#### Proportion of activated CD63^+^/CD203c^+^ basophils

The upregulation of degranulation markers such as CD63 and CD203c is commonly used to express basophil activation upon *ex vivo* stimulation utilizing the area under the curve (AUC) from dose–response curves. Basophil activation correlates with clinical outcomes during OFC and OIT (63, 85).

In 120 PA patients aged 5–55 years from the POISED study, participants were stratified as basophil “non/low,” “intermediate,” and “high” responders based on their baseline BAT (62). Briefly, patients were randomized to receive OIT or placebo. After 2 years of OIT, one group continued with a maintenance dose of 300 mg while the other discontinued for another year (50). Non-responders/low responders tolerated a higher cumulative dose at baseline OFC and experienced the most favorable OIT outcomes. Among the patients who discontinued OIT, 83% and 33% of “non-responders” passed the food challenge to 4 g of peanut protein at 13 and 52 weeks post-therapy, respectively (62). In another OIT study that included 30 patients aged 7–13 years, nine of the 22 patients who were desensitized achieved SU 4 weeks after OIT discontinuation (63). The baseline BAT did not distinguish between these two treatment outcome groups. However, a decrease in the BAT to the major peanut allergen Ara h 2 as early as 3 months of OIT correlated with SU.

#### OIT prediction—infant/toddler vs. later in life

The above-detailed baseline characteristics that might allow the prediction of OIT outcomes have been mainly reported in patients older than 4 years. The first data from the IMPACT trial, targeting infants/toddlers, revealed the involvement of Th2A-related immunotypes. Further studies are needed on this very young age group.

### Baseline humoral profile

Considering the difficulty of stratifying patients based on their “immunotype” in a real-life setting, the utility of serological parameters as markers of treatment outcomes remains an area of interest. In 2017, the DEVIL study confirmed the safety and efficacy of peanut OIT in preschool children younger than 3 years of age. Baseline sIgE and Ara h 2 IgE values were negatively correlated with SU (54). To further evaluate the utility of these serological markers in the same cohort, Dreskin et al. (65) used cut-off values of 35 and 25 kU_A_/L for peanut-sIgE and Ara h 2-sIgE, respectively, to correlate those with the likelihood of achieving SU. To date, only two large randomized control studies have assessed SU to peanut OIT after a prolonged duration of peanut avoidance. Chinthrajah et al. (50) reported that patients over 4 years old who participated in the POISED study with a higher baseline peanut-sIgE/tIgE ratio and Ara h 1 and Ara h 2 IgE titer had a higher risk of treatment failure. Similarly, Jones et al., in a cohort of children from the IMPACT study (≤4 years of age), demonstrated that lower baseline sIgE levels were correlated with the likelihood of “remission” (51).

Table 1 highlights a brief summary of the baseline immune signatures that correlate with peanut OIT outcomes.

## Conclusions

Peanut OIT has proven effective in desensitizing the majority of PA patients and achieving SU in a selected group, which is why it appears to be a promising approach in patient care. However, there are also significant challenges to implementing OIT in real life, where limitations apply to patients (e.g., risk and eligibility profiles to consider) and clinics (e.g., lack of expert centers for frequent visits and OFC). Furthermore, the optimal window of opportunity to start OIT, maintenance dose, frequency, and duration still need to be defined. The IMPACT and DEVIL trials, two recent landmark studies, suggested the optimal timing at the age of less than 3 years. Further studies are needed to compare clinical outcomes in different age groups and dissect underlying endotypes to discover novel surrogate biomarkers predictive of OIT response. In addition to the present findings (Figure 1), further insights might arise from new fields, such as CD8^+^ T cells and the gut microbiome in OIT or blood immune signatures over OFC, contributing to an integrated knowledge base on predictive OIT markers (86–88). This integrated approach will pave the way for precision medicine in food allergy treatment.
